# *Nigella sativa*-chitosan nanoparticles: Novel intestinal mucosal immunomodulator controls and protects against *Salmonella enterica* serovar Enteritidis infection in broilers

**DOI:** 10.1186/s12917-023-03632-1

**Published:** 2023-08-01

**Authors:** Adel Attia M. Ahmad, Gamal A. Elmowalid, Ahmed E. Abdelhamid, Alaa Abed Elrazak Mohammad, Ashraf M. O. Abdelwahab

**Affiliations:** 1 Department of Microbiology, Faculty of Veterinary Medicine, Zagazig University, Zagazig, Egypt; 2 Polymers and Pigments Department, National Research Centre, 33 El-Buhouth St, Dokki, Cairo, Egypt; 3 Faculty of Veterinary Medicine, Zagazig University, Zagazig, Egypt

**Keywords:** Nanoparticles, Chitosan, *Nigella sativa*, Immunomodulation, *Salmonella* Enteritidis, Food-borne diseases

## Abstract

**Background:**

*Salmonella* Enteritidis (SE) propagates in chickens' gastrointestinal surfaces and is transmitted to humans, causing food poisoning. Oral supplementation with natural nanoparticles can overcome the harsh gastrointestinal conditions facing oral vaccines and requires no antibiotic administration to protect against microbial infection. This study was designed to study *Nigella sativa*-chitosan nanoparticles (CNP-NS) prophylactic immunomodulatory efficacy against SE infection in broiler chicks. The CNP-NS was prepared and characterized, and its in *vivo* immunomodulatory activities against an avian virulent-MDR SE-induced challenge in chicks were investigated.

**Result:**

To verify the immune-protective activities of the CNP-NS, colony forming units (CFU) in the liver and fecal droppings; intestinal histopathological alterations and immune cell recruitment; MUC-2, TLR-4, cecal cytokines, and specific IgA gene expression levels were assessed. On the 7th and 12th days after the SE challenge, the CNP-NS supplemented chicks showed complete clearance of SE CFU in livers and fecal droppings, as well as an improvement in food conversion rate compared to non-supplemented CNP-NS that revealed the presence of the challenge SE CFU on the same days. A prominent influx of antigen presenting cells and lymphoid aggregates into the intestinal wall, spleen, and liver was detected with improvements in the intestinal villi morphometry of the CNP-NS-supplemented chicks. The changes of INF-γ, IL-1β, and IL-4 cecal cytokines, as well as TLR-4, MUC-2, and IgA mRNA expression levels, confirm CNP-NS immunomodulatory activities and provide a mechanism(s) for its protective actions against the induced SE challenge of the tested chickens.

**Conclusion:**

These findings suggest promising useful insights into CNP-NS supplementation as a safe food additive for poultry meat consumers' and a protective immunomodulator of the chickens' mucosal immune systems. It could be recommended for epidemiological purposes to reduce the risk of SE food poisoning and transmission to humans.

**Supplementary Information:**

The online version contains supplementary material available at 10.1186/s12917-023-03632-1.

## Introduction

*Salmonella enterica* is one of the most common causes of food-borne illness despite current hygienic measures and is regarded as a serious global public health and economic issue [1]. Contaminated poultry products with non-typhoid *Salmonellae*, especially *S.* Enteritidis (SE), are among the major food-borne diseases causing bacteria in humans. An increase of *Salmonella enterica* serotype Enteritidis infections is linked to poultry products, including meat and eggs, and remains a substantial problem for poultry production. In many countries SE is the most prevalent serovar associated with human salmonellosis and is specifically cited in zoonotic control legislation. The food-borne outbreak of this bacterium is mainly related to the consumption of poultry meat and poultry products from infected, carrier, or fecal contaminated poultry meat during slaughter and other poultry products that have been infected [2–6]. Many poultry species are intestinal carriers, and infection may be carried by feces, fomites, and eggshells, which constitutes a major economic problem worldwide [1]. Many infected birds are culled, and others are rejected at slaughter. The clinical manifestations of *S.* Enteritidis range from self-limiting mild or moderate gastroenteritis to acute systemic infections that lead to mortality in high-risk patients [1]. The development of new alternative strategies is required to control the emergence of pandemic-resistant *Salmonellae* [7].

Control and treatment of enteric infections by antibiotics have multiple disadvantages because of the widespread use or misuse of common antimicrobial agents in veterinary and human medicine for treating and preventing infections [1, 8]. Food-producing animals, particularly poultry, and their products, have become important reservoirs for multidrug resistant bacteria (MDR). Moreover, drug-resistant bacteria, such as SE, can be transferred from these animals to humans through the food chain, thereby limiting the antimicrobial treatment options for severe salmonellosis [9]. Accordingly, healthcare costs have increased due to the increased rate and duration of hospitalization, treatment failure, and death among patients [8]. Protection of poultry flocks against gastrointestinal tract SE infections would limit infection transfer to humans.

In chicken flocks, several vaccination strategies were evaluated against localized pathogenic *Salmonellae* mucosal infections. In contrast, oral and/or injectable vaccines against *S.* Enteritidis infections suffer from drawbacks such as poor protection, injection difficulties, complicated gastrointestinal conditions, mucosal tolerance, elicitation of only cell-mediated immune responses, and failure to stimulate SIgA responses at mucosal surfaces, which is essential for protection against intestinal colonization [10–14]. Vaccinated birds may harbor infection for a number of weeks in the case of live attenuated bacterial vaccines, such as in case of the *Salmonella gallinarum* 9 rough vaccinal strain [15]. In the case of live attenuated *Salmonella gallinarum* 9 rough strain vaccine (SG 9R), vaccinated birds may harbor infection for several weeks due to bacterial persistence in the liver and spleen [15]. Mucosal protection in chickens has met with limited success. To ensure appropriate delivery in the harsh gastrointestinal environment, mucosal delivery techniques including recombinant viral administration have been used [16–18]. The cholera toxin-B subunit elicited very strong SIgA and serum IgG antibody responses and bound efficiently to mammalian intestinal epithelial cell receptors. Oral administration of herbal extracts has been reported to have activity against a wide-ranging spectrum of pathogens [19, 20]. Genetically modified plants encoded with immunizing agents have been tested, and their proteins offer cost-effective edible vaccines. They are specifically designed to provide mucosal activity along with systemic immunity. However, this approach comes with some limitations, which include immune tolerance, unstandardized dosage, denaturation after cocking, and improper preparation [21].

Intestinal mucosal immune responses provide superior protection against disease, but there is currently no FDA-approved adjuvant capable of stimulating humoral and cell-mediated immune responses within mucosal tissues [22]. Antigens encapsulated in ≤ 2 µm microspheres were taken up by most areas of the intestine and induced weak, age-dependent immune responses [23]. Chitosan nanoparticles (CNP), ≥ 2 µm, are potentially useful methods for drug delivery since they are biodegradable, biocompatible, less toxic, and simple to make. Chitosan nanoparticles encapsulate the immunogens efficiently and have positively charged surfaces that facilitate adhesion to host cells with negative charges. Chitosan provides immunogens with slow release [24, 25].

Due to the increasing rates of human food poisoning from consumption of chicken products contaminated with non-typhoidal *Salmonellae* and the economic losses in the poultry industry, it is of paramount importance to find strategies for improving control measures against infection by food-poisoning *Salmonellae* in chickens. The development of natural, safe, and potent oral immunomodulators that could be easily distributed in the harsh gastrointestinal environment and induce mucosal surface protection in chickens is required. The formulation and development of such immunomodulators could control *Salmonella* transmission to humans via meat or poultry mortalities. For centuries, *Nigella sativa* (*N. sativa*) crude oil has been used as an immunomodulator without adverse effects as part of traditional medicine strategies [26]. In this study, we formulated novel oral immunomodulator nanoparticles from chitosan and *N. sativa* (CNP-NS) to be given as a protector to chicks. To the best of our knowledge, this is the first study to propose *N. sativa*-chitosan as a natural immunomodulator nanoparticles against SE infection in broiler chicks to inhibit chicken-to-human SE transfer.

## Materials and Methods

### Bacterial strains' identification

Freshly dead broiler chicks' liver and spleen specimens from ten local broiler producing farms were transferred to the Microbiology Diagnostic Unit, Faculty of Veterinary Medicine at Zagazig University, Egypt, and tested for *Salmonellae* infection. The livers and spleens were processed, then inoculated into nutrient broth and consequently incubated at 37 °C for 24 h. At the end of the incubation period, a loopful of the developed bacterial growth was inoculated into Rappaport broth, incubated at 42 °C for 18–24 h, and then sub-cultured onto XLD for overnight at 37 °C. Colonial growth was identified through inoculation into triple sugar iron agar, examination in motility agar medium, and Rappaport semi-solid Vassiliades [27]. A fresh culture suspension was inoculated into API-E strips. Consequently, the SE identified pure colonies that were kept in BHI with glycerol at -20 °C.

### Nigella sativa crude oil

*Nigella sativa* (NS) crude oil (cold-pressed black cumin oil, Harraz Company for Herbal Products, Egypt) was used in the preparation of chitosan-NS nanoemulsion (CNP-NS).

#### Antibiotics susceptibility testing

SE isolates were tested for antibiotic susceptibility using the disc diffusion method against the following antibiotics: ampicillin (AM10), amoxicillin-clavulanic acid (AMC10/20), ampicillin/sulbactam (SAM10/10), ceftiofur (EFT30), cefotaxime (CEF30), ceftriaxone (CRO30), cefperazone (CFP30), gentamicin (CN10), streptomycin (S10), doxycycline (DO30), ciprofloxacin (CIP5), trimethoprim/sulfamethoxazole (SXT1.25/23.75), chloramphenicol (C30), erythromycin (E15), colistin (CL10), and fosfomycin (FOT200). Imipenem discs were used as control. The data were interpreted according the Clinical and Laboratory Standards Institute guidelines [28]. Each isolate's inhibition zone size and antibiotic resistance profile were reported.

### Preparation of chitosan encapsulated *N. sativa* oil (CNP-NS)

The ionic gelation method was used to create an encapsulated chitosan nanoparticle emulsion [29]. Briefly, chitosan (MWT: 100–300 KDa; Across Co., Ltd., UK) was dissolved in 200 mL of 1% aqueous acetic acid with stirring at room temperature to obtain a 0.5% chitosan solution. To obtain a homogeneous mixture, Tween 80 (1 mL) was added to the chitosan solution and stirred for 30 min. Consequently, NS oil (10 ml) was added to the chitosan 0.5% suspension in a drop-wise manner during homogenization and the ratio of chitosan to NS oil was kept constant at 1:1. Sodium tripolyphosphate (TPP) at a concentration of 0.66 g was dissolved in 100 mL of DW and drop-wise added to the NS-chitosan emulsion under magnetic stirring for 30 min. The solution became turbid upon the addition of the TPP, indicating the production of NS-chitosan nanocapsules (CNP). After CNP-NS formation, the dispersion remained under stirring for an additional hour, then it was removed and stored in the refrigerator at 4 °C until it was used for broiler oral gavage after CNP-NS was characterized.

### Characterization of the CNP-NS

#### TEM (Transmission Electron Microscope)

A transmission electron microscope (HR-TEM, JEOL-JEM-2100) was used to determine the synthesized nanoparticles' size and shape [30]. Before examination, the CNP-NS nanoparticles suspension was sonicated for about an hour in a sonication water bath. The suspension was dropped onto the testing grid (one or two drops) and left to dry in the air prior to the TEM investigation.

#### DLS (Dynamic Light Scattering)

The size and zeta potential of the prepared CNP-NS nanoparticles were measured as previously described [25] by using the Zeta Sizer instrument (Nano-ZS, Malvern Instruments Ltd., UK). The suspension was sonicated to assure good dispersion of the particles in the aqueous medium. Consequently, the DLS of CNP-NS resulting from the Brownian motion of the dispersed nanoparticles emulsion was measured.

### Experimental design, supplementation, colonization breakage and bacterial challenge schedule

Two hundred one-day-old *Salmonellae* free Ross chicks were obtained from Tiba company, kept under hygienic conditions and had ad libitum access to water and antibiotic-free food for 14 days. At 12 days of age, and to further confirm that chicks used in the experiment are *Salmonellae* free, fecal droppings, liver, and spleen specimens were enriched in tetrathionate broth (Neogen, MI, USA) at 37°C for 6 h, and then 10 μL of the enrichment broth was inoculated onto Rappaport–Vassiliadis agar and incubated at 41 °C for 24 h. At the 15^th^ day of the chicks' age, the chicks were subjected to colonization breakage for three successive days by oral gavage of antibiotics cocktail [vancomycin (5 mg/mL), neomycin (10 mg/mL), metronidazole (10 mg/mL), and amphotericin-B (0.1 mg/mL)] every 12 h for three successive days. Consequently, the chicks were randomly distributed into four experimental groups (each with 50 birds): Group I chicks (the CNP-NS supplemented group) were orally gavaged 10 μg of CNP-NS per bird for five days. Group II chicks (the CNP-NS-SE challenged group) received the same oral dose of CNP-NS as Group I, but the chicks were challenged with MDR-SE in BHI at a dose of 1 × 10^9^ per chick after the CNP-NS supplementation. Group III chicks (positive control) did not receive any CNP-NS supplementation, and the chicks were challenged with MDR-SE at the same dose as Group II. Meanwhile, Group IV chicks (negative controls) were given a mock supplemented with 5.0 mL of PBS per chick. The body gain weight (BWG) and food conversion rate (FCR) were calculated. Livers and fecal droppings were collected after 12, 24, 48, 72 h, and at 7 and 12 days of the MDR-SE bacterial challenge for CFU counting. The ceca, ilea, jejuna, livers, and spleens were subjected to histopathological examinations. The fold changes of IgA, IFN-γ, IL-1, TLR-4, IL-4, and MUC-2 mRNA expression levels were determined using RT-qPCR on cecal tonsils collected aseptically in liquid nitrogen. Birds were euthanized in CO2.asphyxiation cage.

### Bacterial count

Bacterial counts per gram of liver and fecal droppings were carried out as previously done [31]. Each sample was prepared under aseptic conditions, serially tenfold diluted in PBS, and then 25 µL of each dilution was spread onto SS agar plates containing ciprofloxacin 1.2 µg/mL and gentamicin 2.0 µg/mL. The plates were incubated at 37°C for 24 h, and the log10 of viable colony counts per gram was calculated. API-E, TSI, motility agar, colony counting, and antibiotic resistance profiles were used to characterize the colonies.

### Histopathology

Specimens from the ceca, ileum, jejuna, duodena, livers, and spleens were collected in 10% neutral buffered formalin during necropsy. Then, 5 µm tissue slices were sectioned and prepared in paraffin and stained with hematoxylin and eosin following a routine protocol before microscopic examinations were performed [32].

### Quantitative real-time PCR

Using quantitative real-time PCR (RT-qPCR), the genes regulating the expression of cytokines (INF-γ, IL-1β, and IL-4); TLR-4; IgA; and Muc-2 in the four experimental groups were analyzed. The relative standard curve of gene expression method was used for quantification of the mRNA expression levels. The gene primers were selected (Table 1), and total mRNA was extracted from cecal tissues. Consequently, mRNA was eluted in RNase-free water and stored at -80 °C until use. The purity of the eluted mRNA was evaluated (A260/A280 ratio, Bio-Photometer; Eppendorf, Hamburg, Germany). For RT-qPCR and the mRNA expression rates of avian cytokines, the amplification cycling condition of each tested gene was done according to the listed references [33–35] (Table 1). The QuantiTect SYBR Green one-step RT-PCR kit (Qiagen) was used according to the instructions of the manufacturer using Mx3000P real-time qPCR equipment (Stratagene, La Jolla, CA) with the cycling conditions of each primer [36] (Table 1). The abundance of each target mRNA was assessed by a 2 − ∆∆CT comparative method [36] with normalization against β-actin in the following formula: − ∆∆CT (sample-control) = (CT of target gene- CT of β-actin gene) sample − (CT of target gene − CT of β-actin gene) control. Results were expressed as a fold change.

**Table 1.**
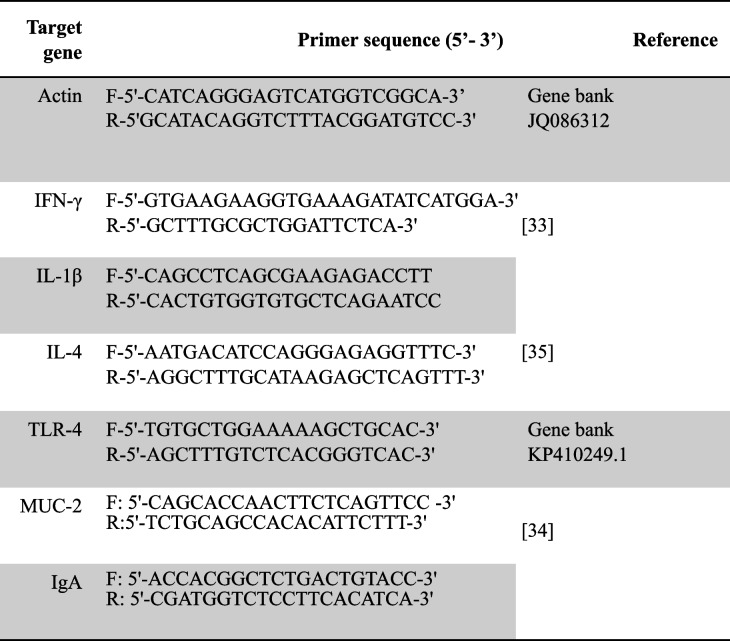
Primers used in real-time quantitative PCR

### Statistical analysis

The data expressed as mean ± standard error mean was analyzed by one-way analysis of variance (ANOVA).

## Results

### Identification of the challenging SE strain

The characteristics of *Salmonellae* isolates from poultry visceral organs were confirmed, and they revealed variable resistance to antimicrobials. The clinically challenging strain was selected based on being MDR (supplementary data). A S. *enterica* serovar Enteritidis clinical isolate with phenotypic resistance markers to ampicillin, amoxicillin-clavulanic acid, ampicillin-sulbactam, ceftiofur, cefotaxime, ceftriaxone, cefperazone, gentamicin, streptomycin, doxycycline, ciprofloxacin, trimethoprim-sulfamethoxazole, chloramphenicol, erythromycin, colistin, and fosfomycin was identified. When pretested by oral challenge with 1 × 10^9^ CFU in BHI broth, the birds displayed gross pathology consistent with *Salmonella* serovar Enteritidis infection. Watery diarrhea, bloody cecal contents, hepatosplenomegaly, and early signs of anorexia were observed post-challenge with the MDR-SE strain. The challenging *Salmonella* serovar Enteritidis strain was reisolated from infected birds, and when identified in TSI, motility agar, and API-E, it showed SE criteria. This clinically MDR-virulent isolate was used in the current study. One MDR-SE virulent isolate was used in the current study.

### *N. sativa*-chitosan-loaded oil nanoparticles (CNP-NS) characterization

**Fig. 1:**
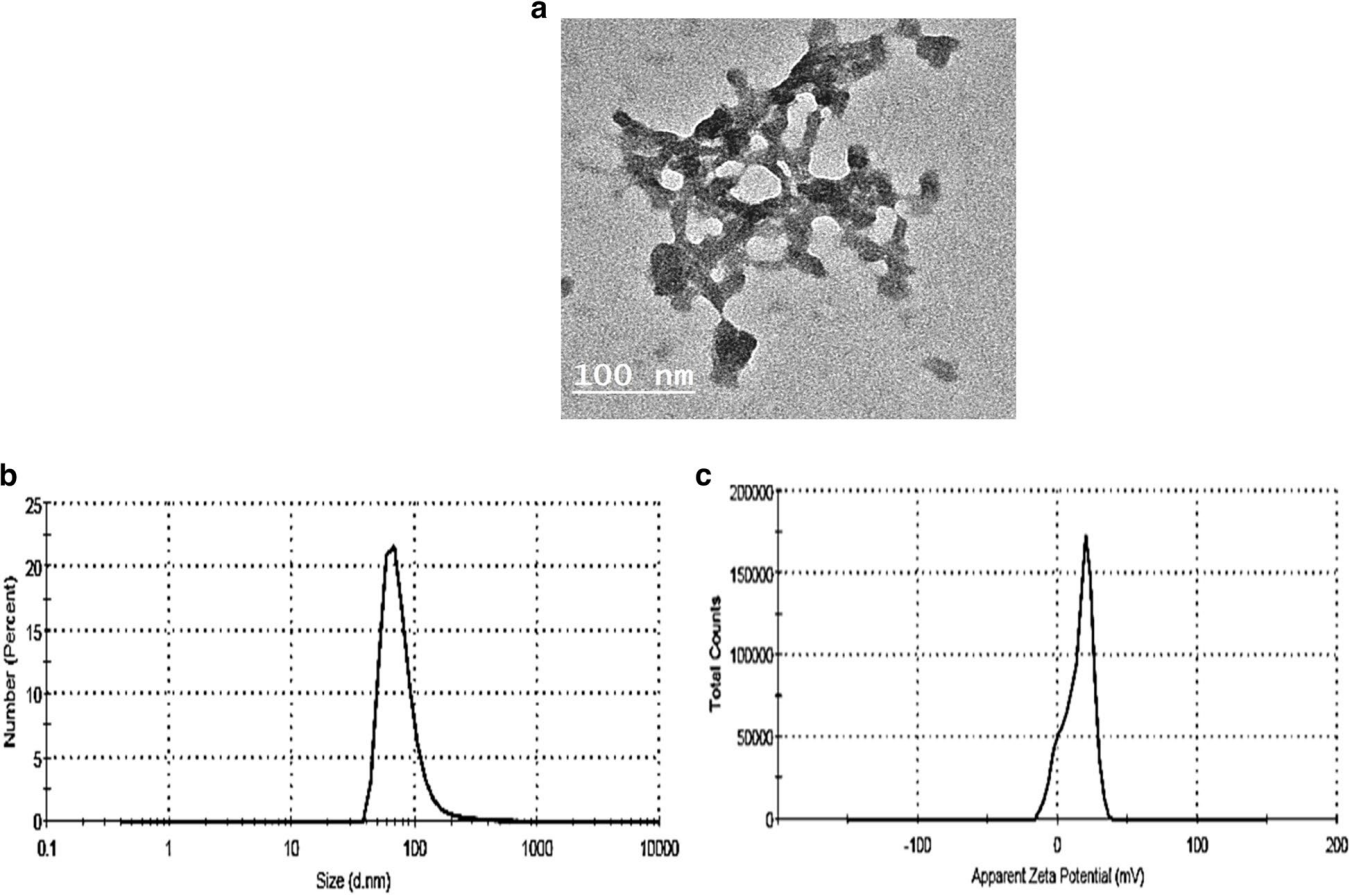
**a** TEM Particle characterization (magnification of 1.0 × 106) before entrapping N. sativa oil. Average particle average size is 100 nm. **b** Particle size distribution of N. sativa-chitosan nanoparticles. **c** Zeta potential of N. sativa -chitosan nanoparticles. The zeta potential was 23.47 mV

- 1. Characterization by a TEM particle size analyzer
  The shape and size of the produced nanoparticles were checked using a TEM particle size analyzer (Fig. 1 a). Chitosan-encapsulated NS nanoparticles were efficiently formulated. The produced particles have a semispherical shape with an average size of about 15–35 nm, with some aggregated and interconnected nanoparticles. Spherical or semi-spherical nanoparticles were observed as a result of the interaction of the positively charged protonated amino groups of chitosan in an acetic acid solution with the negatively charged sodium tripolyphosphate.
- 2. Zeta potential characterization
  The particle size of the prepared nanoparticles was measured using dynamic light scattering (DLS). The average nanoparticle size was 80 nm. Swelling of chitosan nanoparticles in aqueous solution revealed a relatively larger particle size than that of SEM. In addition, the TEM gives an image of the selected area for measurement, while the DLS gives an overall image of the nanoparticles and their aggregations (Figs. 1 b and c).

### Intestinal colonization, invasiveness and shedding of *S.* Enteritidis (SE)

Viable bacterial counts (Log10 SE-CFU/g) of the SE-challenging strain in the chicks' fecal droppings and liver were evaluated. The results revealed a state of bacterial intestinal colonization, SE invasive distribution in both livers, and shedding in fecal droppings. In group III, the non-CNP-NS supplemented (positive control group), at 12 h post-SE challenge, no mortalities were reported, and typical SE infection symptoms such as watery diarrhea, sitting with dropping wings, closed eyes, and loss of appetite were all observed (supplementary data). The challenging SE multiplied progressively, with increasing SE-Log10 CFU/g at 12, 24, 48, 72 h, and at 7 days in both liver and intestine (Table 2), which continued for 12 days post SE challenge. Meanwhile, chicks in group II, the CNP-NS supplemented chicks, showed a gradual decrease of SE-Log10CFU/g in the liver by 1.4 Log10CFU/g at the same intervals, with a complete clearance of SE on the 7^th^ day post-challenge in the liver and fecal droppings (Table 2). SE cultured plates of liver and fecal droppings samples from CNP-NS supplemented and non-supplemented are provided in the supplementary data.

**Table 2:**
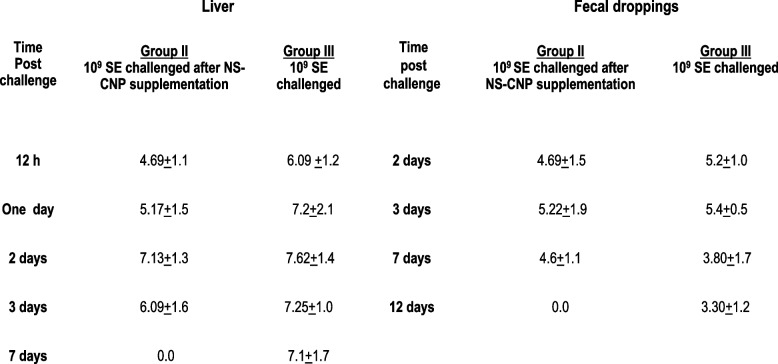
Bacterial counts (CFU mean log 10 value) in liver and fecal droppings

### Food conversion rate

The food conversion rate was assessed in the four experimental groups. Group I supplemented with CNP-NS revealed an improved body gain and food conversion rate compared to those of the control-positive (Group III) and control-negative challenged birds (Group IV), as shown in the supplementary data.

### Pathological views

- 1. Gross lesion
  At 48 h after infection, the sacrificed chicks from Group III (non-CNP-NS supplemented) had watery gut content and congested bowl blood vessels, as well as enlarged congested ceca, liver, and spleen. At day 7 post-challenge, hepatosplenomegaly and a distended gall bladder were observed. Meanwhile, in group II chicks (CNP-NS supplemented), the gut contents were normal and the liver was slightly congested at 48 h without apparent gross lesions in the liver, spleen, or gall bladder at 7 days post-SE bacterial challenge. Gross lesions of the internal viscera and chick showing clinical symptoms of salmonella are provided in the supplementary data.

### Immunohistopathological changes in the gut, liver and spleen at different intervals post *Salmonella* Enteritidis challenge:

**Fig. 2:**
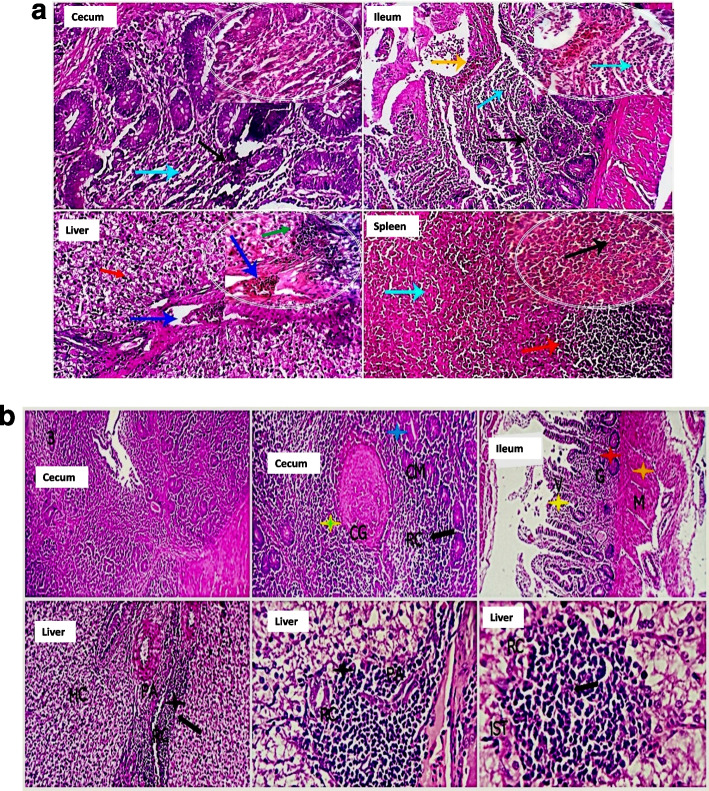
**a**. Histopathology sections of the cecum, ileum, and liver of CNP-NS non-supplemented chicks 24 h after S. Enteritidis- challenge. Marked destructive effects on the cecal, ileal, and jejunal mucosa with the influx of a large number of acute inflammatory cells, including heterophils (light blue arrows). The cecal, ileal, and jejunal glands were partially destroyed by the infiltrated inflammatory cells (black arrows). The villous mucosal epithelium was severely damaged and necrotized. The mucosal blood vessels were moderately hyperemic (orange arrow). Intravascular microthrombosis (blue arrows in the window), moderate round cells and heterophils portal infiltration (green arrow), and hepatocellular degeneration (mostly hydropic degeneration) (red arrow) were all seen in the liver. The spleen demonstrates a mild decrease in the germinal lymphocytic population (red arrow), moderate sinusoidal dilatation, and heterophil infiltration (black arrow), as well as focal histiocytic proliferation (light blue arrow). H&E X 100, 400. **b**. Histopathology sections of the cecum, ileum, and liver of CNP-NS supplemented chicks 24 h after S. Enteritidis-challenge. The histopathological sections show marked influx of lymphocytes (RC, black arrow), while, a few cecal glands appear totally necrotic (CG, green arrow). Ileal tissues have partial epithelial desquamation and mucosal round cells (lymphocytes, plasma cells, and macrophages (yellow and red stars) infiltration. Hepatic tissue shows lymphoplasmacytic infiltrations and aggregations (RC, black arrow), they are also seen in the interstitial tissue without detectable tissue damage (IST) The scale bars are 50 and 100 um

**Fig. 3:**
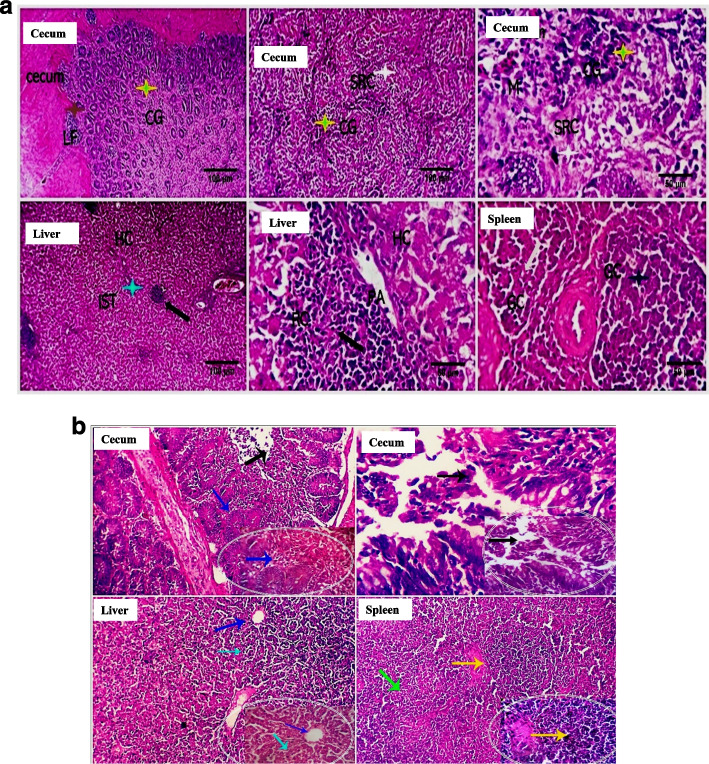
**a** The histopathological changes in the cecum, liver, and spleen of CNP-NS non supplemented chicks at 72 h post-challenge with S. Enteriditis. The cecum demonstrated moderate glandular proliferation (CG, green star), stromal round cell infiltration with replacement of some glands (ST, white star), and regenerative changes in others (CG, green star), lymphoid follicles appear reactive, and some are seen among the muscular walls (LF, brown star). The liver showed portal triads and interstitial lymphoplasmacytic aggregations (PA, RC, IST, light blue star) and apparently normal hepatocytes (HC).The germinal centres (GCs) of the spleen exhibited reactive changes. The scale bars are 50 and 100 um. **b** The histopathological changes in the cecum, liver, and spleen of CNP-NS supplemented chicks at 72 h post-challenge with S. Enteriditis. The cecum show mucosal and submucosal folds, villi, and stromal structures with seemingly normal lining epithelial keeping features, glandular morphology (black arrows), and lymphoplasmacytic inhabitant immune cells (blue arrows). The liver shows apparently normal central veins (blue arrows), portal structures, hepatocytes (light blue arrows) and sinusoidal endothelial Von Kupffer cells. The spleen demonstrates normal histo-morphology of the white pulp germinal centers (yellow arrows), red pulp and splenic cords lymphoplasmacytic populations (green arrows) Stain H&E 100, 400X (windows)

**Fig. 4:**
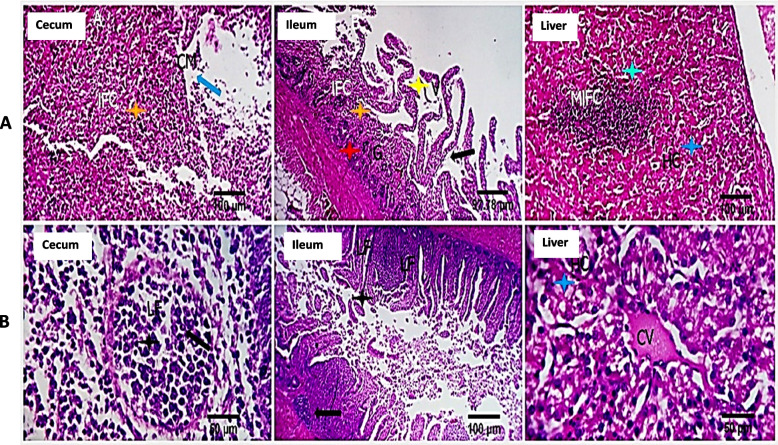
Histopathological changes in the cecum, ileum and liver of CNP-NS non-supplemented (A), and CNP-NS-supplemented chicks (B) at 7 days post challenge with S. Enteriditis. Sections of cecum, ileum and liver collected from CNP-NS non-supplemented chicks post challenge showed characteristic cecal mucosal damage (blue arrow), marked inflammatory cellular infiltration (IFC, orange star). Ileal villous desquamation (yellow star), glandular and crypt destruction (red star) and influx of inflammatory cells (IFC, orange star). Liver with focal necrosis and replacement of necrotic cells by mixed inflammatory cellular aggregates (MIFC, light blue star). Meanwhile sections of cecum, ileum, and liver of the CNP-NS supplemented and then challenged chicks showed cecal immune-reactive lympho-follicular reaction (LF, black star). The ileal payer's patches (LF, lymphoid follicles) contained immune-reactive changes (black star). The liver revealed hepatocellular (blue star) and central vein structures. Stain H&E, Scale bars 50, 100 μm

- **a. Immunohistopathological changes at 24 h post challenge**
  In the non-CNP-NS supplemented chick specimens, the examined histology sections pointed out a marked destructive effect on the cecal, ileal, and jejunal mucosa with the influx of a large number of acute inflammatory cells, including heterophils. In addition, the cecal, ileal, and jejunal glands were partially destroyed by the infiltrated inflammatory cells. The villus mucosal epithelium was severely damaged and necrotized. The mucosal blood vessels were moderately hyperemic. Intravascular microthrombosis, moderate round cells, heterophil portal infiltration, sinusoidal dilatation, Von Kuppfer cell hypertrophy, and hepatocellular degeneration (mostly hydropic degeneration) were all observed in the liver. The spleen revealed mild diminution of the germinal lymphocytic population, moderate sinusoidal dilatation, and heterophil infiltration alongside focal histiocytic proliferation (Fig. 2a). Histology sections of *S.* Enteritidis CNP-NS-supplemented and challenged chicks' cecal tissues revealed focal epithelial denudation, which was associated with a significant influx of lymphocytes that partially replaced the mucosal folds and occasionally aggregated as small lymphoid follicles (Fig. 2b). A few cecal glands were totally necrotic. The ileal villi showed partial epithelial desquamation, mucosal round cells (lymphocytes, plasma cells, and macrophages) infiltration, and mildly dilated blood vessels with perivascular edema. Hepatic tissue represented probable post-vaccination reactive changes, as the hepatocytes in most parts were apparently normal and only a few cells had hydropic degeneration. In most parts of the liver, characteristic delayed hypersensitive immune-reactive lymphoplasmacytic infiltrations and aggregations were encountered; occasionally, they were also seen in the interstitial tissue without detectable tissue damage. The spleen showed apparently normal histomorphology of both white and red pulp, with a normal germinal centers lymphoid population (Fig. 2b).
- **b. Immunohistopathological changes at 72 h post challenge**
  Examined histology sections (Fig. 3a) pointed out a marked destructive effect of the cecal, ileal, and jejunal mucosa with the influx of a large number of acute inflammatory cells, including heterophils in the CNP-NS chick specimens. In addition, the cecal, ileal, and jejunal glands were partially destroyed by the infiltrated inflammatory cells. The villous mucosal epithelium was severely damaged and necrotized. The mucosal blood vessels were moderately hyperemic. Intravascular microthrombosis, moderate round cells and heterophils portal infiltration, sinusoidal dilatation, Von Kuppfer cell hypertrophy, and hepatocellular degeneration (mostly hydropic degeneration) were all observed in the liver. The spleen revealed mild diminution of the germinal lymphocytic population, moderate sinusoidal dilatation, and heterophils infiltration alongside focal histiocytic proliferation (Fig. 3a). Histology sections of *S.* Enteritidis-challenged CNP-NS supplemented chick cecal tissues revealed focal epithelial denudation, which was associated with a remarkable influx of lymphocytes that partially replaced the mucosal folds and occasionally aggregated as small lymphoid follicles (Fig. 3b). A few cecal glands were totally necrotic. The ileal villi showed partial epithelial desquamation, mucosal immune cells (lymphocytes, plasma cells, and macrophages) infiltration, and mildly dilated blood vessels with perivascular edema. Hepatic tissue represented probable post-vaccination reactive changes, as the hepatocytes in most parts were apparently normal and only a few cells had hydropic degeneration. In most parts of the liver, characteristic delayed hypersensitive immune-reactive lymphoplasmacytic infiltrations and aggregations were encountered; occasionally, they were also seen in the interstitial tissue without detectable tissue damage. The spleen showed normal histomorphology of both white and red pulp, with a normal germinal centers lymphoid population (Fig. 3b).
- **c. Immunohistopathological changes in the gut at the 7**^**th**^ **day post-challenge**
  At the 7^th^ day post SE challenge, the cecum, ileum, duodenum, and jejunum showed histopathological views as summarized in Table 3. There was a gradual reversion in villi length, villi width, and crypt length of the ileum to normal lengths in CNP-NS supplemented chicks compared to group CNP-NS non-supplemented chicks, which revealed a reduction in villi size and crypt length with an increase in villi width (Table 3, Fig. 4). Those chicks supplemented with CNP-NS revealed dilated red pulp sinusoids, mucosal aggregates and an influx of heterophils, mature lymphocytes and subsequent aggregates of small lymphoid follicles, as well as remarkable proliferative changes in glandular tissues with mucous secretion and no edema (Table 3 and Fig. 4). In the non-CNP-NS supplemented chicks, there was epithelial desquamation with mucosal damage, mixed inflammatory and lymphatic cell infiltrate; necrotic crypts and glands with dilatation of the blood vessels, and edema (Table 3 and Fig. 4).
- **d. Immunohistopathological changes in the liver at the 7**^**th**^ **day post-challenge**
  The livers of the CNP-NS non-supplemented and then SE challenged chicks showed tissue damage, which included hepatocellular necrosis, biliary involvement, and a few heterophils (Table 4, Fig. 4). Hypersensitive immune responses, tissue damage, as well as heterophils influx in the liver were also observed. Whereas, hydropic degeneration, portal and interstitial delayed hypersensitive immune-reactive lymphoplasmacytic infiltrations and aggregations, and no detectable tissue damage were observed in liver histopathological sections of CNP-NS-supplemented and then SE-challenged chicks (Table 4 and Fig. 4).
- **e. Immunohistopathological changes in the spleen at the 7**^**th**^ **days post-challenge**
  The CNP-NS non-supplemented showed mildly dilated sinusoids in the splenic red pulp with infiltration of many mature lymphocytes and plasma cells and a few heterophils, and the germinal center was reactive. Meanwhile, the splenic tissues of CNP-NS-supplemented chicks showed moderate dilatation of the red pup sinusoids with infiltration of mature lymphocytes and plasma cells and development of a mature germinal center with heterophils influx and diffuse histiocytic reactions (Table 4 and Fig. 4).

**Table 3:**
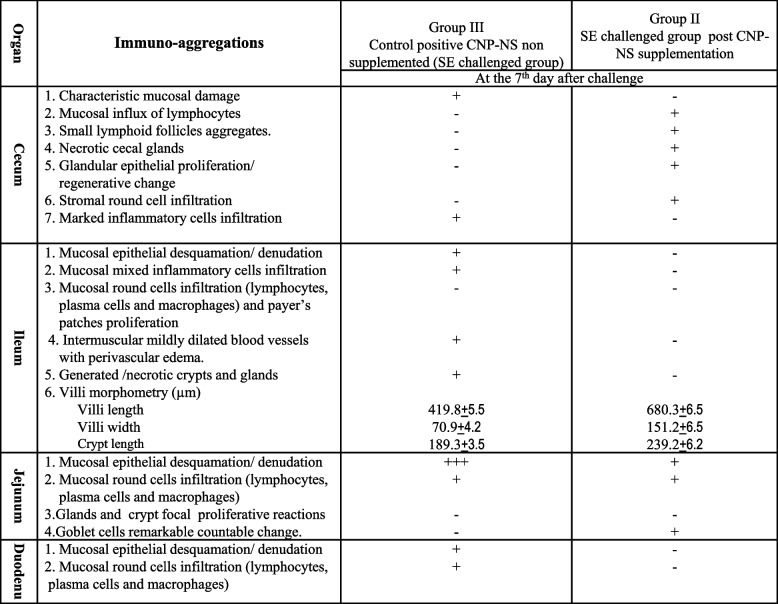
Histopathological changes in the intestine of CNP-NS supplemented and non-supplemented broilers chicks at 7 days post challenge with S. Enteritidis

**Table 4:**
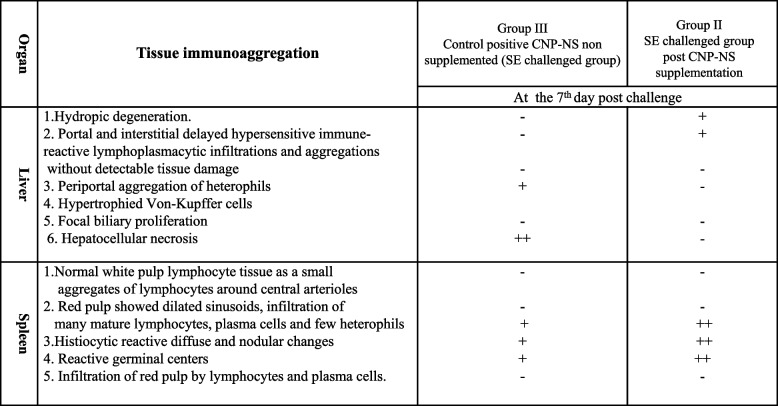
Histopathologyical changes in the liver and spleen in CNP-NS supplemented and non supplemented chicks at the 7th day post SE challenge

### Cecal cytokine mRNA expression findings

**Fig. 5:**
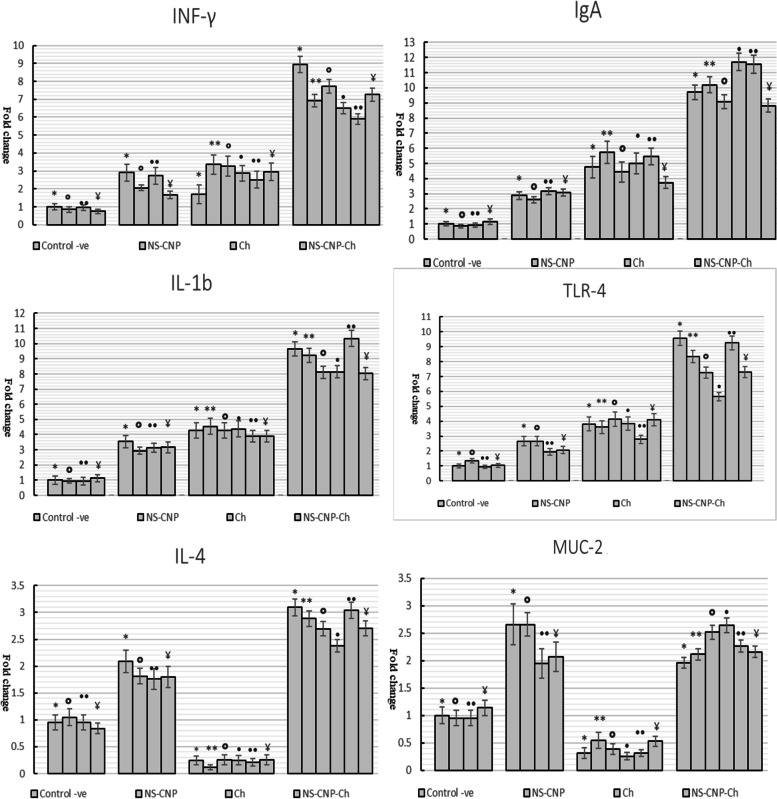
qRT-PCR of IgA, INF-γ, TLR-4, IL-1B, MUC-2 and IL-4 cytokines mRNA expression in the different chicks group. NS-CNP: nanoparticles supplemented group (group I). NS-CNP + CH: nanoparticles supplemented then challenged with SE group (group II). CH: CNP-NS-non supplemented and then challenged with SE (control positive) or group III. Control –ve: non supplemented and mock infected group (group IV). NS-CNP + CH (group II): nanoparticles supplemented then challenged with SE revealed the highest fold change of all examined cytokines followed with detectable drop of fold change in IL-4 and MUC-2 of control positive group (group III). Supplementation *: 12 h, **: 24 h, °: 48 h. •: 72 h, ••: 7 days, ¥: 14 days

At 12, 24, 48, 72 h, and 7 days after SE challenge, experimental groups were examined for transcriptional regulation of mRNA encoding IgA, INF-γ, TLR-4, IL-1, MUC-2, and IL-4. Differences in cytokine mRNA expression were measured as fold-changes with the β-actin gene serving as a control (Fig. 5). The cytokine mRNA expression levels of the CNP-NS supplemented chicks differed from those of the control-negative birds. The CNP-NS supplemented group without the SE challenge (Group I) showed a 7, 5, 7, 2.5, and three fold increase in the mRNA expression of IgA, INF-γ, TLR-4, IL-1β, MUC-2, and IL-4, respectively, compared to the control negative birds. Both IL-4 and MUC-2 levels were lower in the control-positive group (group III) than those in the CNP-NS-supplemented chicks (group I). SE-challenged CNP-NS-supplemented chicks (group II) had higher levels of IgA, INF-γ, TLR-4, and IL-1β (Fig. 5).

## Discussion

Administration of antibiotics as feed additives resulted in the rise of multidrug-resistant bacteria (MDR), so the finding of new safe and effective alternatives is the most up-to-date task for the global community [37, 38]. Herbal product derivatives, as previously reported [39–41], clearly demonstrated in *vitro* safe and potent immunomodulatory activities*. *They can induce non-specific protective immune responses on the gastrointestinal tract's mucosal surface. Clinical trials are required to shed light on the potential mechanism(s) of action of herbal these products. In the current study, we developed natural *N. sativa-*chitosan (CNP-NS) nanoparticle immunomodulators against non-typhoid *Salmonellae*, specifically *S.* Enteritidis (SE). Broiler chicks were used as a natural, validated animal model of SE. When administered orally, CNP-NS nanoparticles improved food conversion rates, demonstrated functional compartmentalization of the chickens' gut mucosal immune system, improved intestinal wall histopathology, induced tissue clearance and inhibited fecal shedding of SE, and modulated the levels of protective mucin, pro-inflammatory, and inflammatory cytokines, as well as immunoglobulin A (IgA). Those improved parameters provided protection against SE-induced infections when clinically tested in broiler chicks.

A virulent clinical MDR-SE strain was used in the current study. The strain revealed resistance to nine antimicrobials representing different antibiotic groups, indicating that those antibiotics could not overcome a natural microbial infection. MDR-SE isolates were frequently isolated [42, 43], indicating the strict need for antibiotic legislation and planning protocols limiting their abuse. Interestingly, oral administration of CNP-NS without antibiotics or other additives was enough to induce protection against a challenge with MDR virulent SE. CNP-NS nanoparticles, when administered in chicks at a low dose per bird (10 μg) for short duration, induced SE clearance in the liver and fecal droppings. In non-CPN-NS-supplemented chicks, the SE strain (10^9^ CFU counts) caused bacterial invasiveness to tissues, and the bacteria remained in the liver and fecal droppings for at least 12 days post-challenge. Previously [44], on the 13^th^ day after the challenge, co-administration of attenuated SE and chicken IFNγ-aided tissue clearance of SE in seven-week layer chicks. In other studies [45–47] and [23], The oral S. Typhimurium vaccine could not efficiently reach the gut-associated lymphoid tissues (GALT) unless preceded by a primary intraperitoneal or subcutaneous immunizing dose to generate protective SIgA in the vaccinated then challenged chickens' intestinal surface. In our study, CNP-NS supplementation reduced the CFU count in tissues and fecal droppings after the virulent MDR-SE induced infection. In a study by [48], higher tissue-invasive CFU counts were reported in one-day old chicks, which may be attributed to the immune system's maturity and the use of different experimental invasive bacterial strains (a mutant of *Salmonella* serovar Typhimurium phage type 14 strain F98). This noticeable drop in CFU of the challenging SE bacteria in the livers and fecal droppings might be due to CNP-NS: (i) extended stability of the CNP-NS; (ii) enhanced immunogenicity; (iii) targeted delivery; and (iv) sustained release [49, 50], and the antimicrobial activity of both chitosan and *Nigella sativa* [50–52]. In *vitro*, adding crude NS oil to monocyte-derived macrophages directly enhanced their size and phagocytic activity and stimulated nitric oxide radical production [39], which might be other causes of chicks' protection. The liver and intestine of CNP-NS supplemented chicks had also a noticeable aggregate and influx of antigen-presenting cells (dendritic and macrophage cells), as well as lymphocytes and germinal centers (GC), which indicate induction of systemic infection and explain the chicks' resistance to the SE challenge.

It is known that food digestion and absorption are crucial functions carried out by the small intestine. The functional integrity of the small intestine requires coordinated specialized features of its different components such as enterocytes, goblet cells and the layer of mucous, resident microflora and immune response. In this study, CNP-NS nanoparticles supplementation improved the intestinal tissues and villi morphology, goblet cell secretions and immune cells localization, according to the obtained histopathological findings. These findings explain the obtained food conversion rate and body gain reported in this study and the other earlier studies [51, 52] after feeding diet containing *N. sativa* and recommend the use of it as a natural growth promotor. Liver and spleen tissue architecture and immune cells recruitment were prominent in gut mucosa. The presence of cecal immune-reactive lympho-follicular reactions and immune-reactive changes in the liver, spleen, and ileal payer's patches and the presence of macrophages and the other antigen-presenting cells (APCs) in the supplemented chicks' tissues indicate immune cell-to-cell interaction to digest and process colonizing SE and to provide protection and reduction of its viable CFU.

The high-molecular-weight mucins protect mucosal surfaces against microbial invasion [53–55]. The mucins layer serves as a physical barrier between the lumen and epithelium that is continually exposed to microbial pathogens [55, 56], confirming the efficacy of the tested nanoparticles in providing mucosal protection. As determined in the current study by histopathological examination and qRT-PCR of the mRNA expression levels of the mucins together with a countable inflammatory response change in CNP-NS-supplemented chicks in contrast to those non-supplemented chicks. Upregulation of mucins mRNA expression could limit the SE colonization and invasion into the intestinal wall and interfere with their adherence to epithelial cells, allowing peristaltic peristalsis to remove the bacteria from the gut lumen [57], and reduce their CFU count.

Toll-like receptors (TLRs) present on immune and epithelial cell surfaces recognize pathogen structure [53]. The TLR-4 expression level of chicks supplemented with CNP-NS was tenfold higher than that of control-negative birds. This expression may be due to the binding of SE cell wall lipopolysaccharide to the TLR-4 receptors expressed in intestinal epithelial cells and the antigen-presenting cells' influxes to associated mucosal lymphoid tissues and to the liver and spleen [56–58]. TRL-4 expression is well known to be associated with the release of inflammatory cytokines such as IL-1β, which activate T helper, which regulates macrophages and the functions of other immune cells [53, 58, 59]. Recruitment of B and T cells and lympho-follicular reaction in CNP-NS supplemented broiler chick visceral tissues and lymphoid organs indicate improved transport through lymphatics and into LNs, as well as the delivery of immunomodulatory molecules to provide systemic immunity that protects the chicks against SE infection. Thymoquinone derivatives of *N. sativa* stimulated TH1 to produce a high level of IFN-γ which is known to stimulate macrophages and natural killer cells to provide protection [52, 60]*.* In a recent study [61], chickens fed with a diet containing 1% *N. sativa* seeds had the highest antibody titers, which may support the 11-fold increase in IgA mRNA expression in the supplemented chicks compared to the control. CNP-NS might be directly involved in B cells differentiating into IgA-producing plasma. The increased mucosal IgA mRNA level observed post-challenge may also be attributed to CNP-NS binding to the APC's surface and induction of co-stimulatory molecule or cytokine expression, as reported [62, 63], which enhances immune cells’ localization in the site of SE invasion and increases their microbial killing activity. The serum bactericidal assay showed phenotypic changes in the SE colonies and significantly reduced SE growth on culture media (supplementary data). Based on our reported data, we hypothesize that enhanced antibody and cytokine responses may emanate from CNP-NS interaction with resident DCs and macrophages that then migrate to the drainage lymph nodes to induce T-cell dependent GC formation and antibody production. Taken together, the data indicated that the administered nanoparticles induce both mucosal and systemic immunity as a result of activated immune cells' migration to both the liver and spleen; GC formation in the cecal tonsils and spleen; and the upregulation of IgA mRNA, TLR-4, mucin, and the immune regulatory cytokines. However, the exact mechanisms of action need to be further investigated.

According to the previous discussion, the proposed mechanisms by which NS-encapsulated conjugated CNPs cause mucosal protection may be due to: i- antimicrobial activity of both chitosan and *N. sativa* oil, ii- the increase in mucins production and inhibition of SE adherence to their receptors; iii- modulating the microflora microenvironment and enhancing their ability to block the mucosal binding sites of pathogenic microbes such as SE, or iv- an upregulation of TRL-4, which increases the cytokines IL-4, IFN-γ, or IL-1β known for their ability to activate macrophages and lymphocytes against SE challenge, as observed in this study. The expansion of GC B-cells, APCs, and antigen-experienced CD4 + T cells following nanoparticle delivery were confined to the intestinal drainage lymph nodes (dLN), suggesting that these responses use intestinal resident immune populations as established by similar-sized nanoparticles (∼200 nm) in the lung that showed resident DC transport to the dLN that, in consequence, resulted in the induction of potent CD4 + T-cell responses [64, 65]. Finally, the promise of CNP-NS does not end with the simple induction of innate and acquired immunity elements, and it could represent a new frontier in the development of personalized immunomodulators. However, studies are needed to examine the intrinsic stability in *vivo*, if there is toxicity, systemic immunity, and, most importantly, the risk–benefit analysis.

## Conclusion

The developed chitosan-loaded *N. sativa* (CNP-NS) nanoparticles enhanced intestinal mucosal immunity and resulted in the protection of broiler chicks against virulent and multidrug-resistant avian* S*. Enteritidis induced infection. Oral administration of CNP-NS nanoparticles to chicks modulated the innate and systemic immunity and resulted in complete clearance of the SE bacterium in the liver and fecal droppings. The CNP-NS preparation was able to recruit immune cells to the intestinal cell wall and improve villi morphometry. The MUC-2 gene is responsible for mucin production, microbial adherence, and colonization prevention in intestinal epithelial cells and was upregulated together with TRL-4, which is responsible for microbial recognition and cytokine production signaling in the supplemented chicks. Cecal cytokines (INF-γ, IL-4, and IL-1β) and IgA gene expression were also found to be modulated. These improved parameters, when combined, resulted in improved broiler chick protection against SE infection. We recommend the use of CNP-NS in broiler chicks for prophylactic and therapeutic purposes against SE broiler infections as an alternative to antibiotic usage.

## Supplementary Information

**Additional file 1. **

## Data Availability

The datasets used and/or analyzed during the current study available from the corresponding author on reasonable request.
