# Supplementary material for: Nigella sativa-chitosan nanoparticles: Novel intestinal mucosal immunomodulator controls and protects against Salmonella enterica serovar Enteritidis infection in broilers
Source: BMC Vet Res. 2023 Aug 1;19:103. doi: 10.1186/s12917-023-03632-1 (PMC10391840; doi:10.1186/s12917-023-03632-1)
Supplement: Supplementary file 1 — Additional file 1. [file 12917_2023_3632_MOESM1_ESM.ppt]

## Slide 1
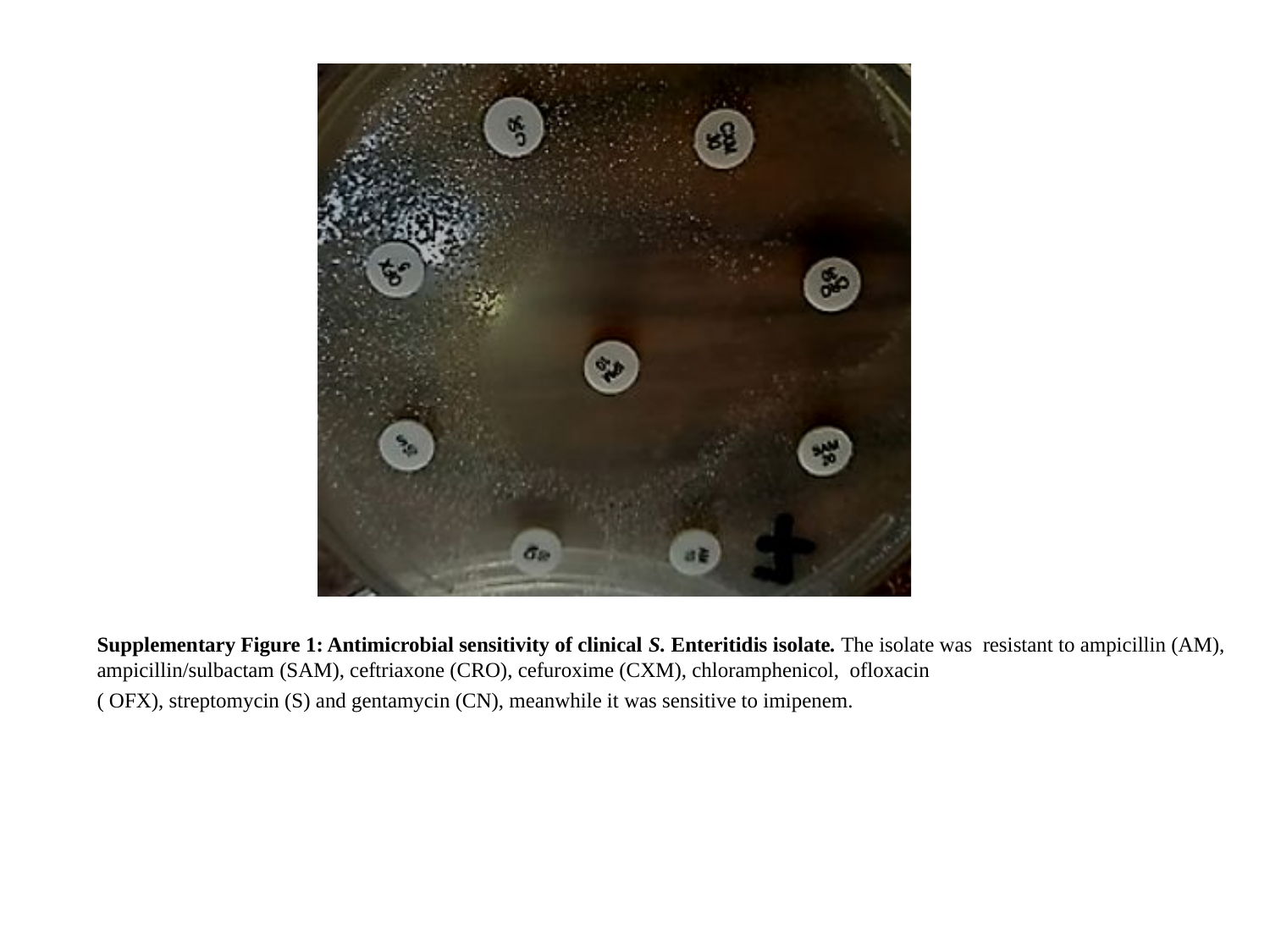

Supplementary Figure 1: Antimicrobial sensitivity of clinical S. Enteritidis isolate. The isolate was resistant to ampicillin (AM), ampicillin/sulbactam (SAM), ceftriaxone (CRO), cefuroxime (CXM), chloramphenicol, ofloxacin
( OFX), streptomycin (S) and gentamycin (CN), meanwhile it was sensitive to imipenem.

## Slide 2
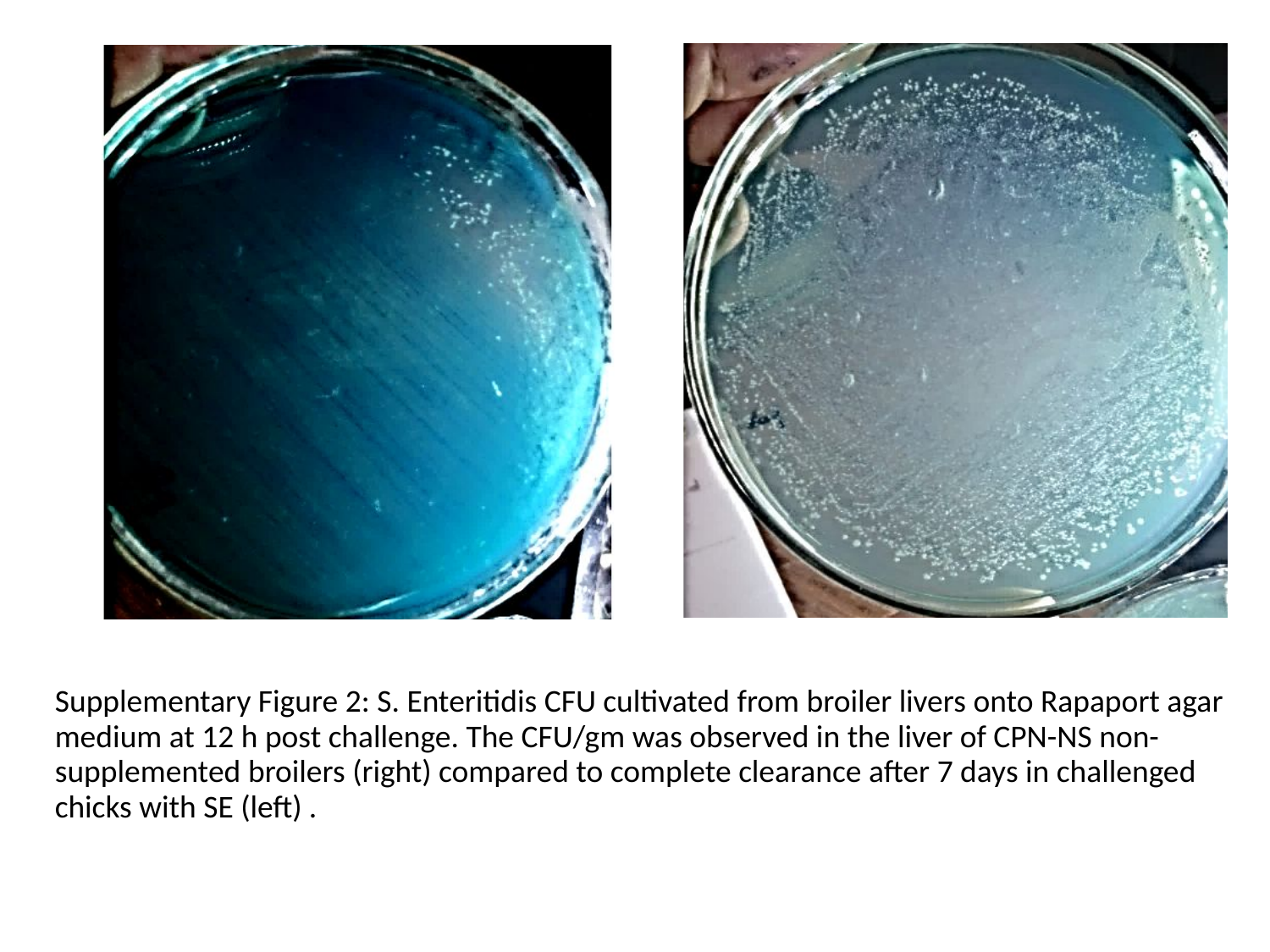

Supplementary Figure 2: S. Enteritidis CFU cultivated from broiler livers onto Rapaport agar medium at 12 h post challenge. The CFU/gm was observed in the liver of CPN-NS non-supplemented broilers (right) compared to complete clearance after 7 days in challenged chicks with SE (left) .

## Slide 3
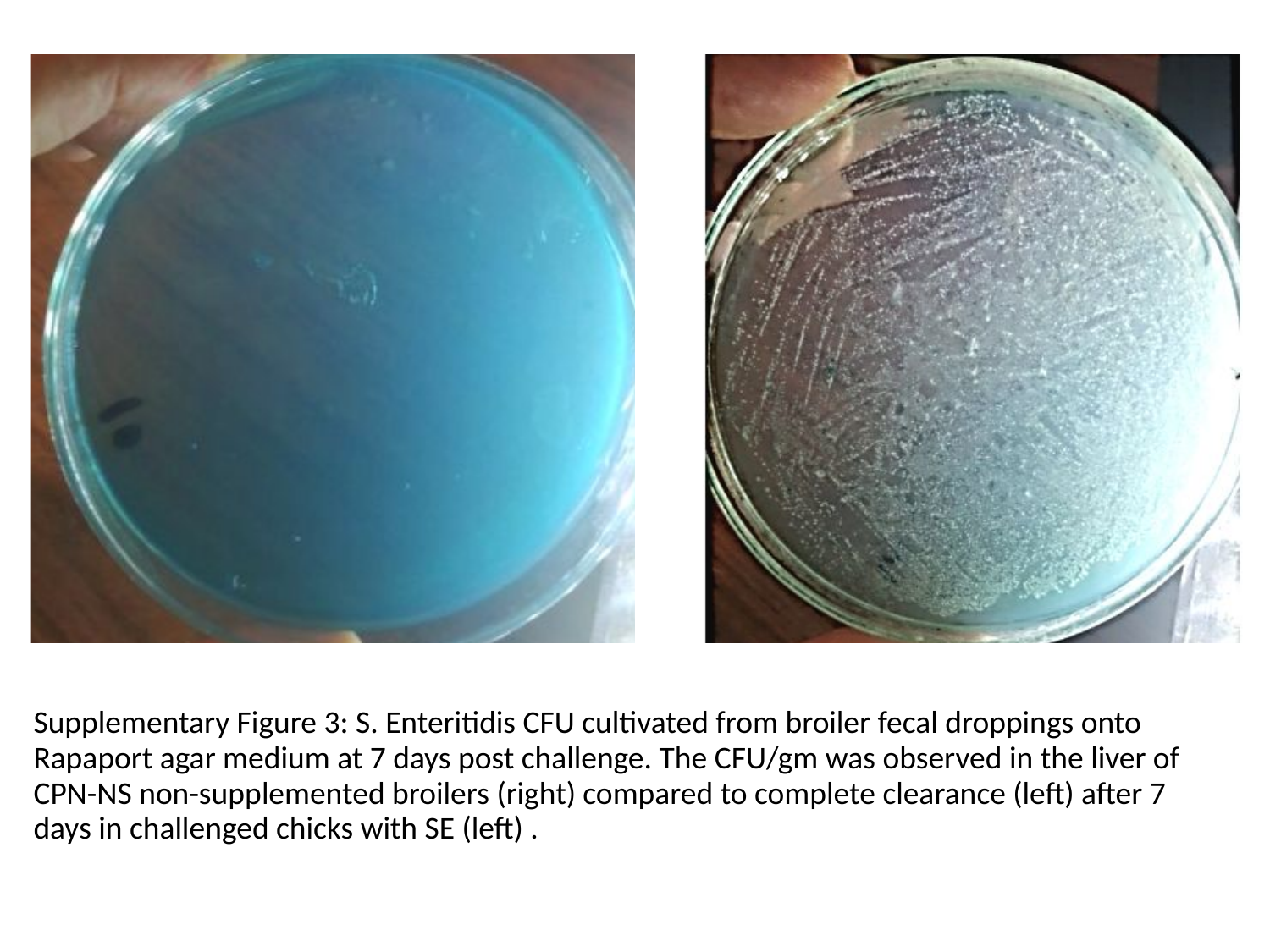

Supplementary Figure 3: S. Enteritidis CFU cultivated from broiler fecal droppings onto Rapaport agar medium at 7 days post challenge. The CFU/gm was observed in the liver of CPN-NS non-supplemented broilers (right) compared to complete clearance (left) after 7 days in challenged chicks with SE (left) .

## Slide 4
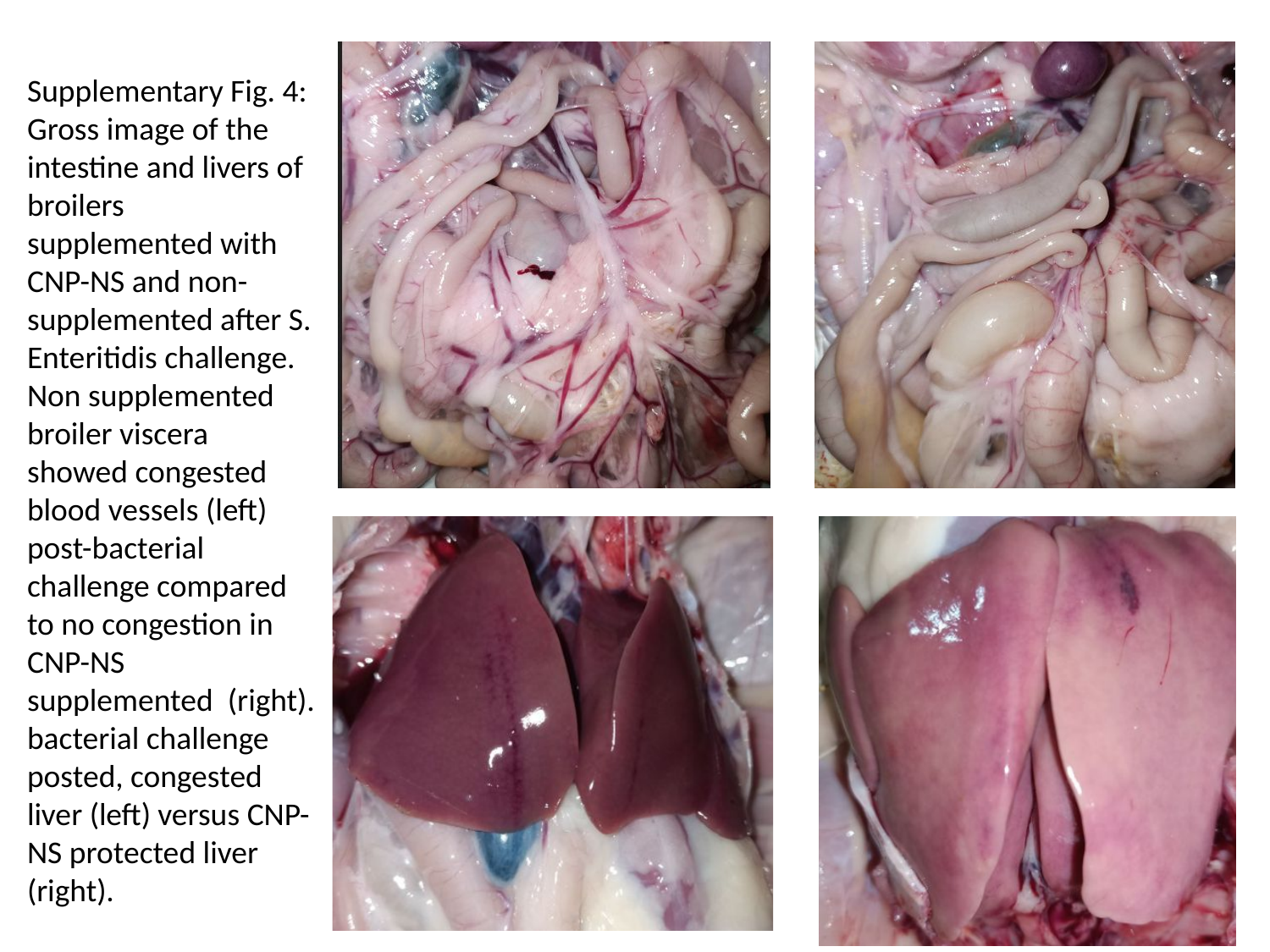

Supplementary Fig. 4: Gross image of the intestine and livers of broilers supplemented with CNP-NS and non-supplemented after S. Enteritidis challenge. Non supplemented broiler viscera showed congested blood vessels (left) post-bacterial challenge compared to no congestion in CNP-NS supplemented  (right). bacterial challenge posted, congested liver (left) versus CNP-NS protected liver (right).

## Slide 5
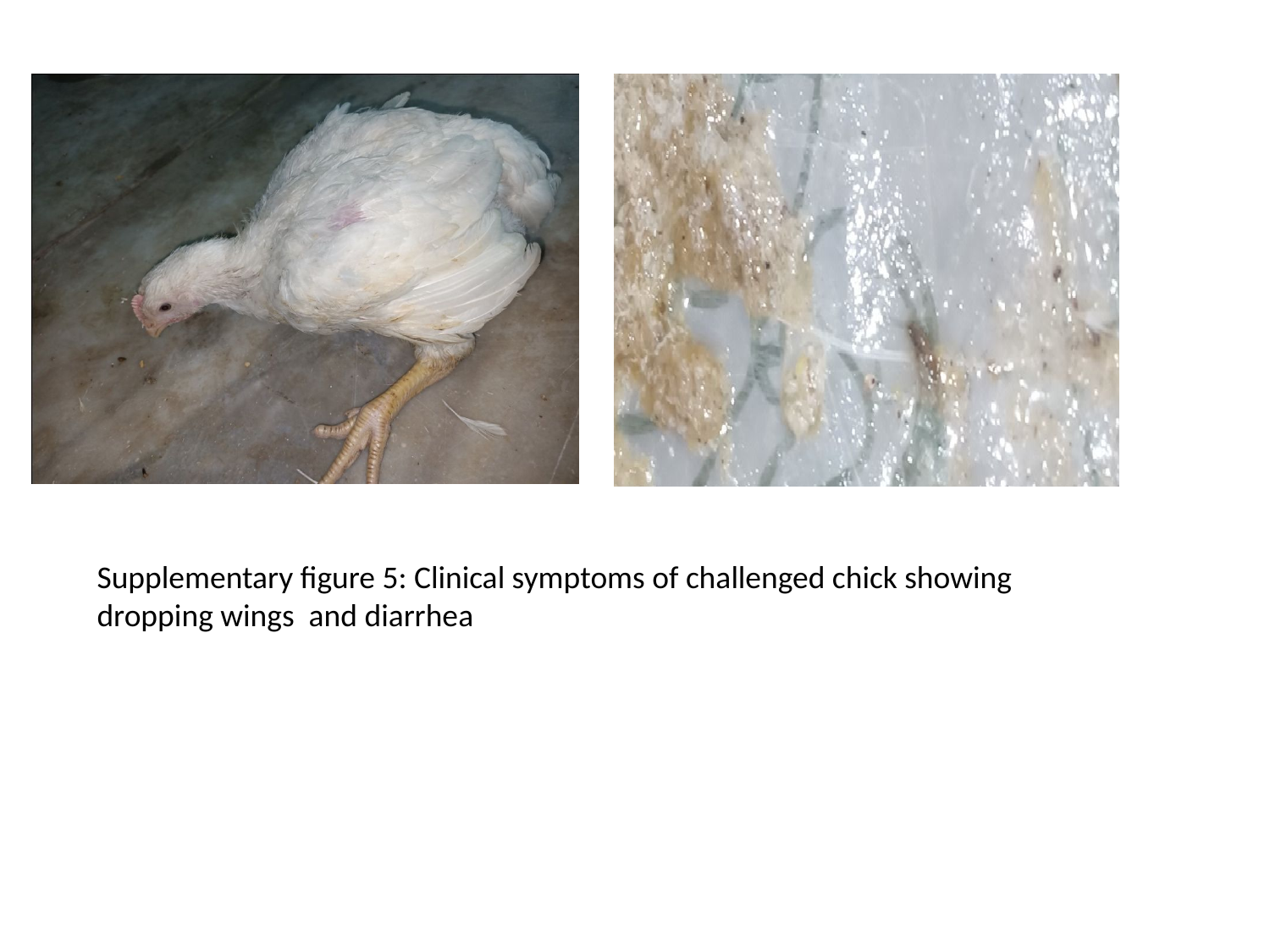

Supplementary figure 5: Clinical symptoms of challenged chick showing dropping wings and diarrhea

## Slide 6
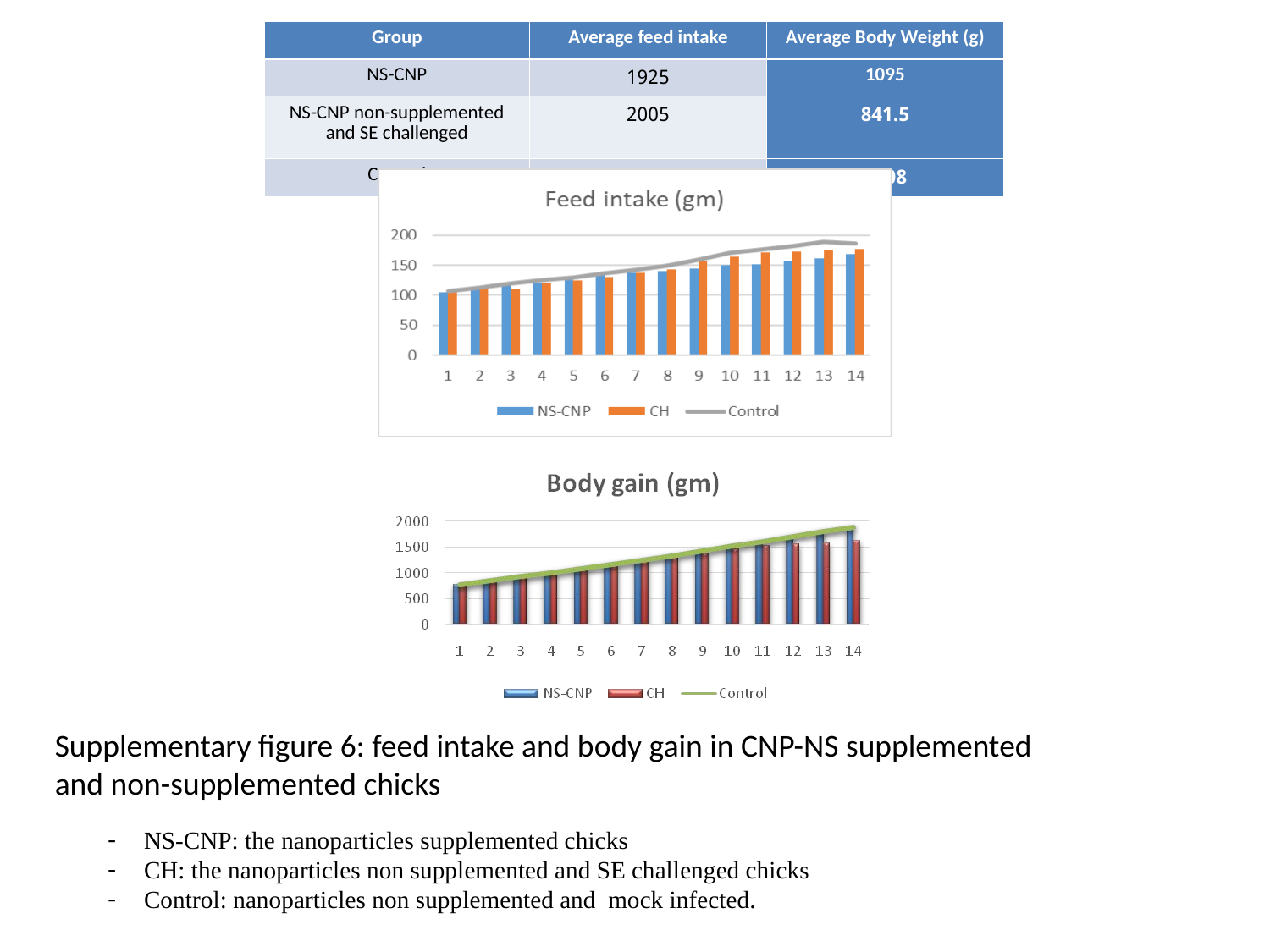

| Group | Average feed intake | Average Body Weight (g) |
| --- | --- | --- |
| NS-CNP | 1925 | 1095 |
| NS-CNP non-supplemented and SE challenged | 2005 | 841.5 |
| Control | 2084 | 1108 |
Supplementary figure 6: feed intake and body gain in CNP-NS supplemented
and non-supplemented chicks
 NS-CNP: the nanoparticles supplemented chicks
 CH: the nanoparticles non supplemented and SE challenged chicks
 Control: nanoparticles non supplemented and mock infected.

## Slide 7
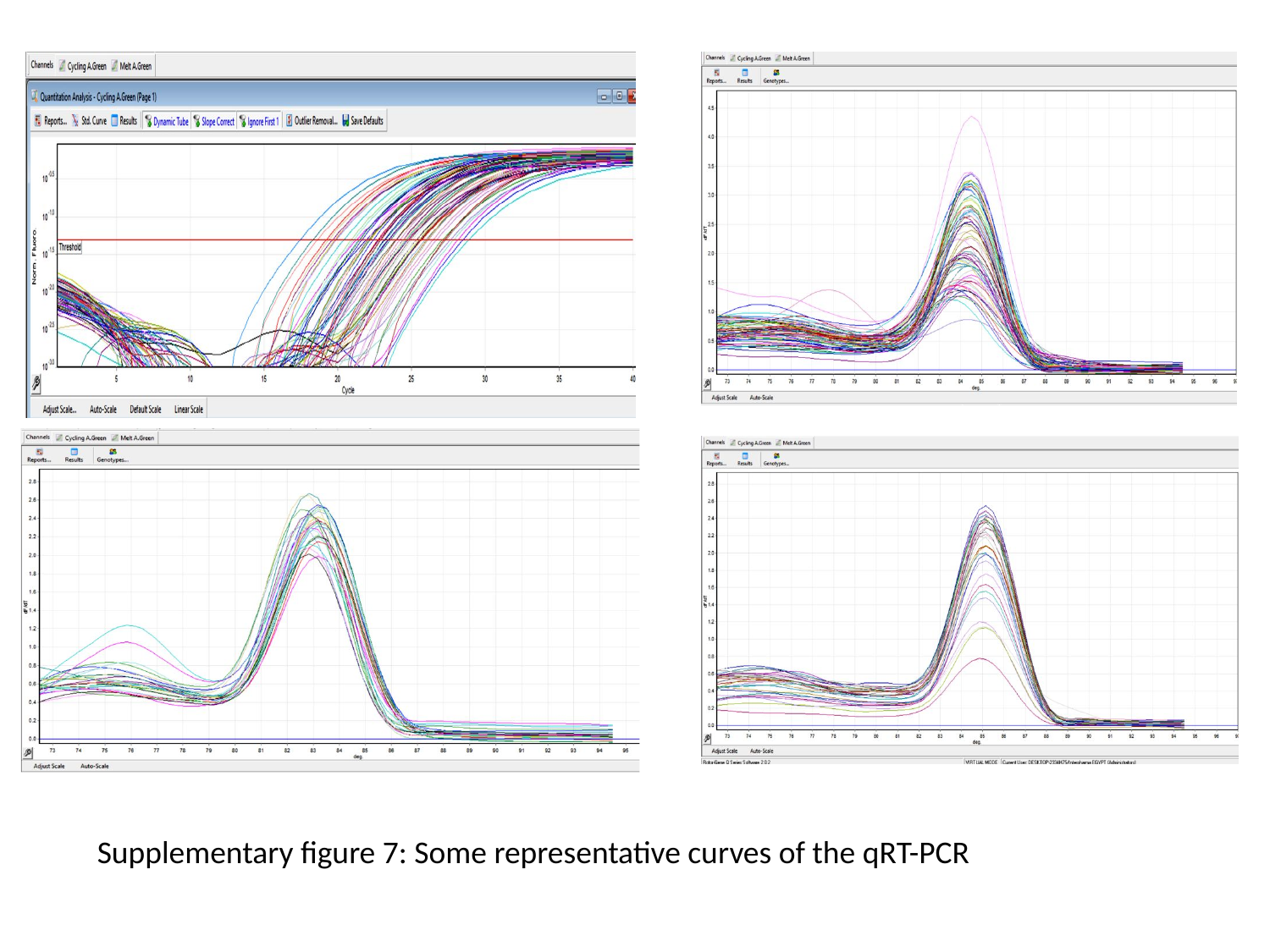

Supplementary figure 7: Some representative curves of the qRT-PCR

## Slide 8
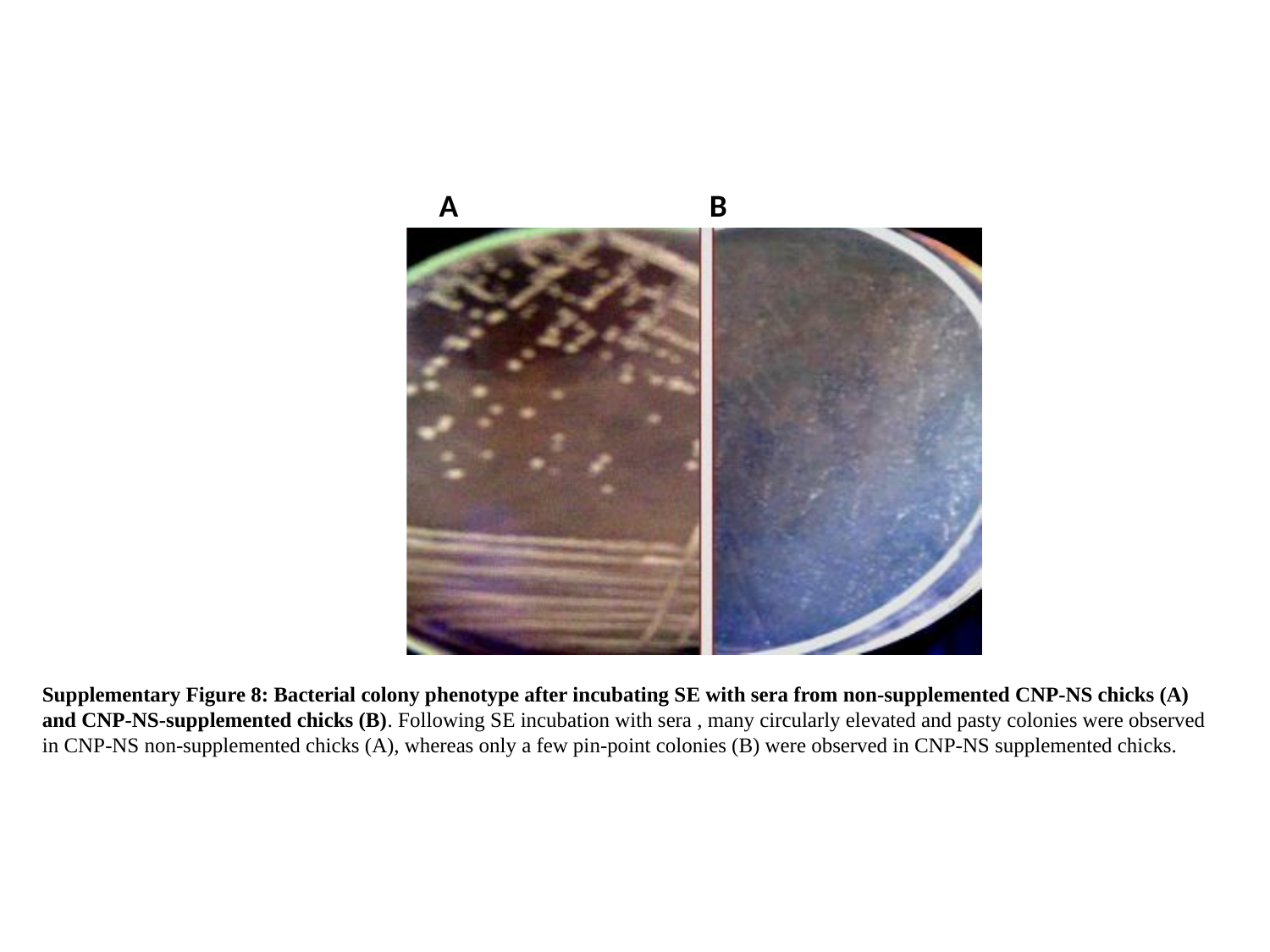

A B
Supplementary Figure 8: Bacterial colony phenotype after incubating SE with sera from non-supplemented CNP-NS chicks (A) and CNP-NS-supplemented chicks (B). Following SE incubation with sera , many circularly elevated and pasty colonies were observed in CNP-NS non-supplemented chicks (A), whereas only a few pin-point colonies (B) were observed in CNP-NS supplemented chicks.
